# Relationship between Serum Selenium Level and Self-Reported History of Kidney Stone

**DOI:** 10.3390/nu15112549

**Published:** 2023-05-30

**Authors:** Anni Wang, Ningrui Wang, Dongfeng Zhang, Jing Wen, Weijing Wang

**Affiliations:** 1 Department of Epidemiology and Health Statistics, School of Public Health of Qingdao University, 308 Ningxia Road, Qingdao 266071, China; wangan199682@126.com (A.W.); zhangdongfeng@qdu.edu.cn (D.Z.); 2 School of Public Health, Ningxia Medical University, Yinchuan 750004, China; wangnrui111036@outlook.com

**Keywords:** kidney stone, selenium, nutrition surveys

## Abstract

A relationship may exist between selenium and kidney calculi, but there is a lack of research in this field at present. Our study explored the relationship between the serum selenium level and a medical history of adult kidney calculi. We utilized data from the National Health and Nutrition Examination Survey conducted between 2011 and 2016. Participants self-reported their history of kidney stones, while serum selenium levels were measured using inductively coupled plasma dynamic reaction cell mass spectrometry. Our findings indicate a negative correlation between serum selenium levels and the risk of kidney stone history. In the multiple-adjusted model, the lowest serum selenium level group had a higher risk than the other groups. The odds ratio (95% confidence interval) of ever having kidney stones for the highest serum selenium level group was 0.54 (0.33–0.88). In the results of stratified analysis, this relationship was still significant in the groups of women and those 40–59 years. We also found that as a nonlinear dose–response relationship between serum selenium levels and the history of kidney stones disease. In our research, we found that people with higher serum selenium levels had a lower risk of having a history of kidney stones. We concluded that selenium may have a protective effect on kidney stones. In the future, more population studies are needed to explore the relationship between selenium and kidney stones.

## 1. Introduction

Based on an analysis of data from the National Health and Nutrition Examination Survey (NHANES) spanning from 2007 to 2018, it was found that approximately 12% of men and 10% of women in the United States had a prior history of kidney stones [1]. Kidney stones have imposed a huge economic burden on health care, and they affect people’s quality of life [2,3]. Current studies have found a variety of kidney-calculi-related factors, including genetic factors [4] and environmental factors [5] such as sex [6], obesity [7], and personal habits [8]. There is also an interaction between common chronic diseases, such as diabetes and hypertension, and kidney calculi [9]. Among the factors affecting kidney stones [10,11], nutritional factors play an important role [12,13,14]. At the same time, the relationship between trace elements and kidney calculi was also studied. Previous studies have investigated the correlation between kidney stones and dietary factors, including the consumption of caffeine [15], vitamin C [16], vitamin A [17], pyridoxine [18], zinc [19], and copper [20]. The relationship between the serum levels of various substances and kidney calculi, such as serum calcium, potassium, sodium [21], albumin [22] and magnesium [23], has also been studied.

Selenium is a crucial trace element for the human body and plays a vital role in various biological processes [24,25]. As a cofactor, it participates in the regulation of various enzymes and has many functions, such as regulating thyroid hormone metabolism [26] and antioxidation [27]. Among them, the selenium concentration in the thyroid is the highest. Selenium is widely present in the natural environment, including the air and soil. The primary dietary sources of selenium include bread, cereals, eggs, meat, fish, fruits, and vegetables [28]. Excessive or insufficient selenium intake is harmful to human health [29]. Previous studies have found that selenium might be related to coronary heart disease [30], type 2 diabetes mellitus [31], nonalcoholic fatty liver disease [32], thyroid diseases [33], and other diseases [34,35]. In addition, the relationship between selenium and cancer has also been widely studied [36].

Previous studies have evaluated the relationship between selenium and kidney stones in animal models. One study, which involved 48 Wistar rats, found that selenium has the ability to inhibit the formation of stones [37]. Additionally, a separate study of 20 male dogs demonstrated that selenium supplementation reduced the formation of calcium oxalate kidney stones induced by ethylene glycol [38]. Two studies have explored the relationship between dietary selenium intake and kidney calculi. One study studied the elderly and found that the dietary selenium intake of adults over the age of 60 was negatively correlated with kidney calculi [39]. Another study focused on participants aged 0–80 years old and found a negative correlation between dietary selenium intake and kidney calculi risk, especially for young people (<50 years old), men, and those with overweight/obesity (body mass index ≥ 25.0) [40]. In addition to dietary selenium intake, people might also obtain selenium from supplements, so the relationship between serum selenium level and kidney calculi has some research value [41].

At present, there is a lack of research on the relationship between serum selenium and kidney calculi. The association between serum selenium levels in adults and the incidence of kidney stones has not yet been investigated, and the dose–response relationship has not been evaluated, so we used the research data from the NHANES from 2011 to 2016 to explore the relationship between them.

## 2. Materials and Methods

### 2.1. Study Population

The National Health and Nutrition Examination Survey (NHANES) provided us with the necessary research data, including basic demographic characteristics, questionnaire information, and laboratory data. This program consisted of multiple surveys that focused on various health topics and populations. The survey results have been crucial in determining the prevalence and risk factors of diseases [42]. Many studies have conducted cross-sectional studies using NHANES data and have obtained some valuable findings.

**Figure 1:**
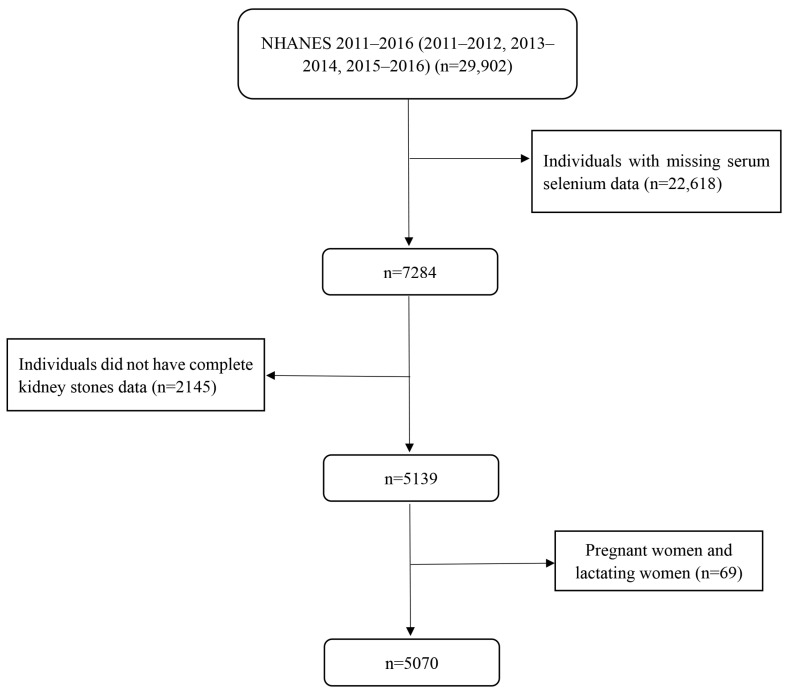
Flowchart for selecting qualified participants from the NHANES.

We used the data of three NHANES cycles (2011–2012, 2013–2014, and 2015–2016), which included serum selenium level data. At the beginning, the total number of people was 29,902. In 2011–2016, NHANES randomly selected one-third of the subsamples aged 6 years and above to measure the serum selenium level and randomly selected subsamples aged 20 years and above to be used in a questionnaire survey to obtain kidney disease data. In order to ensure the integrity of participants’ research data, our study excluded participants lacking serum selenium data and lacking kidney stones data. In addition, pregnant and lactating women also were excluded from our study [43]. Finally, our research included 5070 participants over the age of 20 years, and a detailed flow chart can be found in Figure 1.

### 2.2. Assessment of Kidney Stones

The prevalence of kidney calculi, an outcome variable, was obtained through a questionnaire survey. In the kidney condition section of the questionnaire data [44], there was a question “Have you ever had kidney stones?” If the participant answered yes, they were considered to have a history of kidney stones disease [45]. If they answered no, there was no history of kidney calculi disease.

### 2.3. Evaluation of Serum Selenium Level

Serum selenium levels were obtained from laboratory data. The serum selenium level was measured via inductively coupled plasma dynamic reaction cell mass spectrometry (ICP-DRC-MS). Samples were stored in a suitable freezing condition (−20 °C) and transported to the National Center for Environmental Health for testing. The three cycles of data had the same lower detection limit. The lower limit of detection (LLOD) of serum selenium was 4.5 (g/dL) [46]. The serum selenium level was divided into four groups (Q1, Q2, Q3, and Q4), according to quartile [47].

### 2.4. Covariates

We considered several variables, including social and economic status, health habits, and health factors, along with dietary variables such protein and calcium consumption, in connection with kidney stones to eliminate factors potentially confounding the research outcomes [16,19,20]. The data were collected through two 24 h retrospective interviews and questionnaires [48], and a detailed classification information of covariates is presented in Table 1, including some continuous and categorical variables.

### 2.5. Statistical Analysis

To ensure greater representativeness in the complex sampling survey design, it is imperative in NHANES data analysis to utilize accurate weights. According to the analytic notes of NHANES, we used the specific sample weight of the serum selenium data in the data file [49]. The characteristics of the participants were compared and analyzed. The normality of the continuous variables was tested using the Kolmogorov–Smirnov test. or Normally distributed variables are expressed as mean ± standard deviation (SD), and Student’s *t*-test was used to compare characteristics between the groups of those who had ever had kidney stones and those who had not. Non-normally distributed variables are described as the median (interquartile range), and the Mann–Whitney U test was used for comparison.

Stata 15.0 (Stata Corp., College Station, TX, USA) was used to perform the primary statistical analysis. The lowest serum selenium group (Q1) was taken as the reference group. Logistic regression analyses were used to explore the relationship between serum selenium and kidney stones. We report the results as odds ratio (OR) and 95% confidence interval (95% CI). Model 1 adjusted for age, sex, education level, marital status, PIR, race, smoking, drinking, physical activity, BMI, hypertension, stroke, and diabetes. Based on model 1, model 2 added an adjustment for the dietary intake of protein, water, calcium, magnesium, potassium, and sodium. When the *p*-value was less than 0.05, the results were considered statistically significant. Restricted cubic spline was used to explore the dose–response relationships between serum selenium and kidney stones. The three nodes were located at the 5th, 50th, and 95th percentile of serum selenium [50]. To investigate the correlation between serum selenium levels and kidney calculi, our research also included stratified analysis of sex and age.

## 3. Results

Finally, 5070 participants were included in the study, and their average age was 49.15 ± 17.51 years old. Among them, 490 people had a history of kidney stones, accounting for 9.66% of the total. Table 2 shows a comparison of the results of the characteristics between ever had kidney stones (yes) group and ever had kidney stones (no) group. The data in the categorical variable table are expressed as the number of participants (weighted percentage), and the continuous variable is expressed as the median (interquartile range).

There were differences between the two groups in some baseline characteristics. People who had ever had kidney stones were more likely to have a higher BMI, lower recreational activity, and lower dietary intakes of magnesium and potassium. Smokers, hypertension, and diabetes were more likely to have a history of kidney stones.

In the crude model, we did not find any correlation between the serum selenium level and kidney stones. The study found that in model 1, individuals with the highest serum selenium level had an odds ratio of 0.58 (95% confidence interval: 0.38–0.90) for ever having kidney stones compared with those with the lowest serum selenium level. In model 2, which adjusted for multiple factors, the odds ratio (95% confidence interval) for the highest serum selenium level group was 0.54 (0.33–0.88) compared with the lowest serum selenium level group. The joint test of the effect for the multiple categorical variables was used; the serum selenium level was negatively correlated with kidney stones, with an OR value of 0.984, *p* = 0.015, and the confidence interval was 0.972–0.997. The results of the two models were consistent, and the results are presented in Table 3.

Table 4 shows the correlation between serum selenium and kidney stones after sex stratification. In women, the odds of ever having kidney stones were lower in the group with the highest serum selenium level, with an odds ratio of 0.53 (95% confidence interval of 0.30–0.93) compared with the group with the lowest serum selenium level. In men, the association between serum selenium levels and kidney calculi was no longer significant.

Table 5 shows the correlation between serum selenium levels and kidney stones after age stratification. For those 40–59 years of age, compared with the lowest serum selenium level group, the odds ratio (95% confidence interval) of the ever having kidney stones group for the three serum selenium level quartiles group was 0.36 (0.19–0.69). Other age groups showed no significant association.

**Figure 2:**
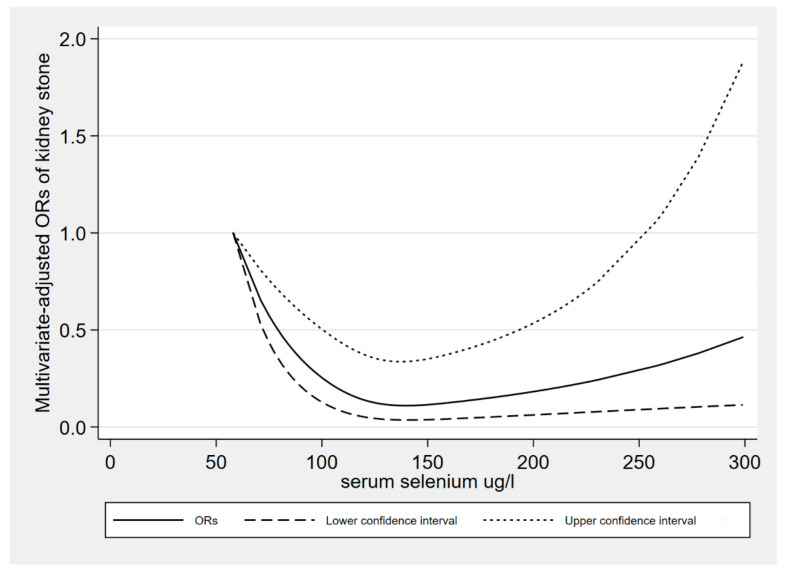
The dose–response relationship between serum selenium level and kidney stones.

This study found a nonlinear correlation between the serum selenium level and kidney stones (P *_for nonlinearity_* = 0.0003), as shown in Figure 2. Consuming an appropriate level of serum selenium had a protective effect on the presence of kidney calculi. The risk of kidney stones was found to be relatively low when serum selenium level was around 137.5 ug/L. The results of the restricted cubic spline data showed that when the serum selenium level was 230 ug/L, the OR value was 0.24, and the 95% confidence interval was 0.08–0.74; when the serum selenium level was 258 ug/L, the OR value was 0.31, and the 95% confidence interval was 0.09–1.06. Some results are shown in Table 6.

## 4. Discussion

To the best of our knowledge, this is the first study to evaluate the serum selenium level and the history of kidney stones disease in adults. Our analysis showed that in the general population, the serum selenium level was negatively correlated with the risk of kidney stone disease history. There was a small difference between the lower bound of Q4, 139 ug/L, and 137.5 ug/L; Q3 vs. Q1 was close to a meaningful result. The OR value was 0.67, and the confidence interval was 0.42 to 1.08. This resulted in no significant differences between Q2 vs. Q1 and Q3 vs. Q1, but significant differences were observed between Q4 vs. Q1. In the analysis of sex stratification, the negative correlation between serum selenium level and the risk of kidney stone history was still significant among women. After age stratification analysis, we found that the negative correlation between the serum selenium level and the disease history of kidney stones in the 40–59 years age group was still significant. We also found a nonlinear dose–response relationship between serum selenium level and the history of kidney stones disease. According to the results in Figure 2, it was found that when the serum selenium level was about 137.5 ug/L, the risk of kidney calculi was relatively low. At present, the mechanism between selenium and kidney calculi is not very clear. In the past, some animal experiments explored the relationship between selenium and kidney calculi. The protective effect of selenium on kidney calculi may be achieved as follows. In an animal experiment on 20 male dogs, the formation of stones was induced by ethylene glycol aqueous solution, and it was found that sufficient selenium reduced the formation of kidney calculi of calcium oxalate in dogs [38]. It was pointed out that the downregulation of osteopontin expression induced by selenium might be the mechanism through which the formation of kidney stones is inhibited in dogs. A study of 48 rats found that selenium can significantly reduce the deposition of calcium oxalate, which might be due to its inhibition of the aggregation and growth of new crystals [37]. Another study on rats also found that dietary vitamin E and selenium supplementation can reduce the risk factors of the urinary system, prevent lipid peroxidation in tissues, and inhibit oxalate synthesis [51]. Free radical reactions damage renal tubules and promote the formation of calcium oxalate crystals [52]. Selenium enhances the body’s antioxidant and free-radical-scavenging abilities, and reduces the oxidative damage of active oxygen to the kidney [53]. Based on the information from previous studies that were referenced, the activity would be mainly focused against papillary monohydrate calcium oxalate renal stones, avoiding or protecting against the intrapapillary lesions that induced the formation of such stones [52,54]. In addition, two cross-sectional studies using NHANES data found a negative correlation between dietary selenium intake and kidney calculi [39,40]. Both studies targeted the U.S. population but had different age ranges for participants. Participants in the two studies were 6669 elderly participants and 184,240 general people, respectively. This finding is in accordance with that in our research, which suggests the protective effect of selenium on kidney calculi.

There are several advantages to our research. Firstly, the dose–response relationship between serum selenium levels and kidney stone disease was explored. Secondly, in multiple regression analysis, we adjusted the dietary intake and other confounding factors. Thirdly, this study explored the relationship between gender and age stratification. Fifthly, the variable in our study was serum selenium level, which reflected the selenium intake of diet and supplements. Compared with diet survey, it better reflected the selenium level of the human body. Finally, the outcomes were deemed more dependable due to the extensive sample size.

Our research also has certain limitations. Firstly, it was difficult to determine the causal relationship in cross-sectional research. Second, there might have been a wrong classification in collecting outcome variables through questionnaires. Thirdly, 24 h dietary recall can cause memory bias, which could have affected how our research outcomes were estimated. Fourthly, there might have been other unadjusted confounding factors. Fifthly, the incidence rate in kidney calculi was obtained through a questionnaire survey, which might have contained some errors. Finally, the results from the restricted cubic spline suggest that excessively high serum selenium levels might no longer be protective. Among our subjects, only seven subjects had serum selenium levels above 230 ug/L, which may have resulted in inaccurate upper limit results, and the specific upper limit value needs more participants for further exploration in the future.

## 5. Conclusions

According to our research, there is a link between serum selenium levels in the general population and the likelihood of having kidney stones in the past, and selenium may have a certain protective effect on kidney calculi, which provides some enlightenment regarding the prevention of kidney calculi. The relationship between selenium and kidney stones needs to be further researched through population studies.

## Figures and Tables

**Table 1:** The detailed classification information of covariates.

Classification	Covariates
No; Yes	Smoker ^a^, drinker ^b^, hypertensive ^c^, diabetic ^d^, stroke patient ^e^
Continuous	Protein, water, calcium, magnesium, potassium, and sodium intake (mg/day)
Vigorous ^f^; moderate ^g^; other	Occupational and recreational physical activity
Mexican American; other Hispanic; non-Hispanic White; non-Hispanic Black; other race	Race
20–39; 40–59; ≥60	Age (year)
≤0.99; and ≥1	Poverty–income ratio (PIR)
Male; female	Sex
Married/living with partner; widowed/divorced/separated/never married	Marital status
≤25; 25 to <30; ≥30	Body mass index (BMI) (kg/m^2^)

^a^ Smoked at least 100 cigarettes in life. ^b^ Had at least 12 alcohol drinks/year. ^c^ Ever been told by a doctor or other health professional that they had hypertension, or currently taking antihypertensive drugs, or systolic blood pressure (SBP) ≥130 mm Hg, or diastolic blood pressure (DBP) ≥80 mm Hg. ^d^ Ever been told by a doctor or other health professional that they had diabetes or were taking insulin or fasting plasma glucose (FPG) level at least 126 mg/dL, or 2 h plasma glucose level above 200 mg/dL or a glycated hemoglobin (HbA1c) level of 6.5% or greater. ^e^ Self-reported physician diagnosis of stroke ^f^ Work involves vigorous-intensity activity that causes large increases in breathing or heart rate such as carrying or lifting heavy loads, digging, or construction work for at least 10 min continuously. ^g^ Work involves moderate-intensity activity that causes small increases in breathing or heart rate such as brisk walking or carrying light loads for at least 10 min continuously.

**Table 2:** Participants’ baseline traits according to whether they had a history of kidney stones, NHANES 2011–2016 (N = 5070).

Trait	Ever Had Kidney Stones (No)	Ever Had Kidney Stones (Yes)	*p*-Value
Number of participants (%) ^a^	4580 (90.34)	490 (9.66)	
Age (year) ^a^			0.0006
20–39	1639 (36.71)	104 (21.87)	
40–59	1521 (36.95)	170 (43.52)	
≥60	1420 (26.34)	216 (34.61)	
PIR (%) ^a^			0.4001
<1	923 (13.91)	117 (15.56)	
≥1	3657 (86.09)	373 (84.44)	
Sex (%) ^a^			0.0334
Male	2249 (47.95)	285 (55.67)	
Female	2331 (52.05)	205 (44.33)	
Material status (%) ^a^			0.5484
Married/living with partner	2885 (66.45)	310 (68.42)	
Widowed/divorced/separated/never married	1667 (33.55)	176 (31.58)	
Educational level (%) ^a^			0.1549
<High school	970 (15.17)	126 (18.54)	
High school	956 (19.88)	106 (20.70)	
>High school	2478 (64.95)	246 (60.76)	
Race/ethnicity (%) ^a^			0.0001
Mexican American	629 (8.79)	62 (5.85)	
Other Hispanic	489 (6.31)	65 (6.21)	
Non-Hispanic White	1705 (65.30)	245 (75.93)	
Non-Hispanic Black	1028 (11.42)	63 (5.82)	
Other races	729 (8.18)	55 (6.19)	
BMI (kg/m^2^) (%) ^a^			0.0013
<25	1381 (30.51)	109 (21.79)	
25 to <30	1453 (33.32)	154 (31.40)	
≥30	1688 (36.17)	223 (46.81)	
Work activity (%) ^a^			0.6106
Vigorous	839 (20.97)	89 (22.65)	
Moderate	923 (22.31)	85 (20.16)	
Other	2814 (56.72)	315 (57.19)	
Leisure activity (%) ^a^			0.0151
Vigorous	1106 (27.56)	76 (18.90)	
Moderate	1165 (27.68)	125 (30.82)	
Other	2306 (44.76)	289 (50.28)	
Alcohol consumption (%) ^a^	2960 (78.42)	345 (79.85)	0.5554
Smoker (%) ^a^	1929 (42.86)	253 (53.04)	0.0036
Stroke (%) ^a^	157 (2.51)	24 (2.38)	0.8284
Diabetes (%) ^a^	810 (17.69)	162 (33.06)	<0.0001
Hypertension (%) ^a^	2482 (50.57)	320 (62.76)	0.0002
Serum selenium (μg/L) ^b^	128.05 (21.6)	127.8 (22.1)	0.9501
Water intake (gm/d) ^b^	2468.75 (1261)	2380 (1201)	0.3692
Protein intake (gm/d) ^b^	74.445 (41.49)	72.145 (41.65)	0.2648
Calcium intake (mg/d) ^b^	822.25 (562.5)	826.5 (508.5)	0.4579
Magnesium intake (mg/d) ^b^	276 (152)	259 (157.5)	0.0162
Potassium intake (mg/d) ^b^	2473 (1255)	2381 (1200.25)	0.0334
Sodium intake (mg/d) ^b^	3136.5 (1758.5)	3135.5 (1569)	0.6083

^a^ Chi-square test was used for comparison. ^b^ Mann–Whitney U test was used for comparison.

**Table 3:** The weighted odds ratio (95% confidence interval) for history of kidney stones according to quartiles of serum selenium levels.

	Cases/Participants	Crude ^a^	Model 1 ^b^	Model 2 ^c^
Serum selenium (μg/L)				
Q1 (<118.1)	126/1276	1.00 (ref)	1.00 (ref)	1.00 (ref)
Q2 (118.1 to <128.1)	121/1261	0.97 (0.75–1.26)	0.82 (0.56–1.20)	0.78 (0.51–1.21)
Q3 (128.1 to <139.7)	122/1272	0.97 (0.75–1.26)	0.74 (0.48–1.13)	0.67 (0.42–1.08)
Q4 (≥139.7)	121/1261	0.97 (0.75–1.26)	0.58 (0.38–0.90) *	0.54 (0.33–0.88) *

^a^ Unweighted. ^b^ Adjusted for race, age, marital status, sex, educational level, BMI, work or recreational physical activity, PIR, alcohol consumption, smoking status, diabetes, hypertension, and stroke; being weighted. ^c^ Based on model 1, the adjustment for dietary moisture, calcium, sodium, magnesium, potassium, and protein intake was added. * *p* < 0.05; being weighted.

**Table 4:** Association between serum selenium and kidney stones after sex stratification.

Serum Selenium	Cases/Participants	Odds Ratio ^a^	95% CI	*p*-Value
Men				
Q1 (<118.1)	63/515	1	1	
Q2 (118.1 to <128.1)	66/600	0.826	0.41–1.65	0.581
Q3 (128.1 to <139.7)	75/663	0.881	0.48–1.62	0.680
Q4 (≥139.7)	81/756	0.596	0.31–1.14	0.115
Women				
Q1 (<118.1)	63/761	1	1	
Q2 (118.1 to <128.1)	55/661	0.770	0.45–1.31	0.327
Q3 (128.1 to <139.7)	47/609	0.499	0.24–1.03	0.059
Q4 (≥139.7)	40/505	0.528	0.24–1.03	0.059

^a^ Adjusted by the weighted analysis required by NHANES; adjusted race, marital status, age, educational level, BMI, work or recreational physical activity, PIR, alcohol consumption, smoking status, diabetes, hypertension, stroke, and dietary water, calcium, sodium, magnesium, potassium, and protein intakes.

**Table 5:** Association between serum selenium level and kidney stones after age stratification.

Serum Selenium	Cases/Participants	Odds Ratio ^a^	95% CI	*p* Value
20–39 years old				
Q1 (<118.1)	28/487	1	1	
Q2 (118.1 to <128.1)	30/468	0.724	0.35–1.49	0.347
Q3 (128.1 to <139.7)	27/406	1.068	0.46–2.47	0.875
Q4 (≥139.7)	19/382	0.494	0.24–1.03	0.059
40–59 years old				
Q1 (<118.1)	43/430	1	1	
Q2 (118.1 to <128.1)	40/397	0.899	0.44–1.83	0.765
Q3 (128.1 to <139.7)	43/450	0.364	0.19–0.69	0.003
Q4 (≥139.7)	44/414	0.585	0.27–1.27	0.172
≥60 years old				
Q1 (<118.1)	55/359	1	1	
Q2 (118.1 to <128.1)	51/396	0.757	0.38–1.49	0.412
Q3 (128.1 to <139.7)	52/416	1.239	0.59–2.61	0.564
Q4 (≥139.7)	58/465	0.657	0.31–1.40	0.271

^a^ Adjusted by the weighted analysis required by NHANES; adjusted race, marital status, sex, educational level, BMI, work or recreational physical activity, PIR, alcohol consumption, smoking status, diabetes, hypertension, stroke, and dietary water, calcium, sodium, magnesium, potassium, and protein intake.

**Table 6:** The results of restricted cubic spline data.

Serum Selenium (ug/L)	Odds Ratio	95% CI
75	0.57	(0.44–0.76)
85	0.41	(0.27–0.64)
95	0.30	(0.16–0.55)
105	0.22	(0.10–0.46)
115	0.16	(0.06–0.40)
125	0.12	(0.04–0.35)
135	0.11	(0.04–0.34)
145	0.11	(0.04–0.34)
155	0.12	(0.04–0.36)
166	0.13	(0.04–0.39)
175	0.14	(0.05–0.42)
187	0.16	(0.06–0.47)
197	0.18	(0.06–0.52)
207	0.19	(0.07–0.57)
218	0.22	(0.07–0.65)
224	0.23	(0.08–0.69)
230	0.24	(0.08–0.74)
258	0.31	(0.09–1.06)
262	0.33	(0.10–1.12)
278	0.38	(0.10–1.39)
298	0.46	(0.11–1.85)

## Data Availability

In this study, publicly accessible datasets were analyzed. This information can be found at: https://wwwn.cdc.gov/nchs/nhanes/continuousnhanes/default.aspx (accessed on 7 March 2023).
